# Assessment of pharmacokinetic parameters, oral bioavailability, and physiological effects of amantadine in horses

**DOI:** 10.1186/s12917-026-05672-9

**Published:** 2026-06-23

**Authors:** Hannah L Riley, Khursheed R Mama, Megan E Jacobs, Daniel S McKemie, Philip H Kass, Heather K Knych

**Affiliations:** 1 K. L. Maddy Equine Analytical Chemistry Laboratory (Pharmacology Section), School of Veterinary Medicine, University of California, Davis, CA USA; 2 Department of Clinical Sciences, Colorado State University, Fort Collins, CO USA; 3 Department of Population Health and Reproduction, School of Veterinary Medicine, University of California, Davis, Davis, CA USA; 4 Department of Molecular Biosciences, School of Veterinary Medicine, University of California, Davis, CA USA

**Keywords:** Pain, NMDA receptor, Multi-modal, Pharmacodynamics, Dose response

## Abstract

**Background:**

Without adequate pain management, central nervous system plasticity can result in acute or adaptive pain developing into chronic or maladaptive pain. This is difficult to manage and often unresponsive to traditional analgesics. An important mechanism associated with maladaptive pain is central sensitization which results in part from increased NMDA (N-methyl-D-aspartate) receptor activation. Amantadine is an NMDA receptor antagonist that has been used in combination with opioids and NSAIDs in humans and dogs for the treatment of conditions associated with central sensitization. The goal of the current study was to describe the pharmacokinetics and physiological effects of amantadine in horses as a first step in assessing the utility of this drug in horses. Eight horses received a single intravenous administration of 5 mg/kg and oral administrations of 3, 5, and 10 mg/kg of amantadine in a randomized 4-way balanced crossover design. Blood samples were collected before drug administration and at predetermined time intervals for up to 96 h post-drug administration. Concentrations of amantadine were determined using liquid chromatography-tandem mass spectrometry and pharmacokinetic analysis was performed. Behavioral assessments (e.g., sedation, ataxia), locomotor activity, gastrointestinal borborygmi, and effects on heart rate were assessed.

**Results:**

The C_max_ and T_max_ of amantadine were 208.7 ± 70.4, 347.9 ± 79.5, and 478.9 ± 165.6 ng/mL and 0.63 (0.16–0.75 h; median and range) 0.63 (0.16–1.0 h), and 1.0 (0.5–1.0 h) for 3, 5, and 10 mg/kg, respectively. The terminal half-life was 3.90 ± 1.24 following IV administration of 5 mg/kg, and 3.87 ± 2.17, 4.21 ± 1.41, and 4.87 ± 2.05 h following oral administration of 3, 5, and 10 mg/kg, respectively. A 2-compartment population PK model best described concentration data. The absorption rate constant was 3.16 1/h and the oral bioavailability was 23.2%. Transient signs, including ataxia and hypometria were observed in 5/8 horses following IV administration.

**Conclusion:**

Amantadine administration to horses was well tolerated, supporting additional study. This study provides pharmacokinetic and limited safety data that can be used to guide pharmacodynamic studies to assess the therapeutic efficacy of this compound in horses.

**Supplementary Information:**

The online version contains supplementary material available at 10.1186/s12917-026-05672-9.

## Introduction

Effective pain management in horses is challenging due to subtle clinical signs, side effects of analgesic drugs, and the complexity of treating maladaptive pain. Early recognition of pain and treatment with multimodal therapies including regional anesthetics, non-steroidal anti-inflammatory drugs (NSAIDs) and opioids can help minimize behaviors associated with pain which foster a return to normal function [1–3]. If pain management is inadequate, central nervous system plasticity can result in acute or adaptive pain developing into chronic or maladaptive pain [4, 5].This progression is known as the wind-up phenomenon, where prolonged noxious input causes sensitization of the central nervous system [4, 5]. As a result, noxious signals become amplified and previously non-noxious stimuli become noxious [4, 5]. An important mechanism of central sensitization is increased NMDA (N-methyl-D-aspartate) receptor activation within the dorsal horn of the spinal cord [6]. Management of wind-up pain in horses is anecdotally less responsive to analgesics including NSAIDs and opioids. Furthermore, long-term administration of NSAIDs and opioids is associated with significant adverse effects, including gastroduodenal ulcers and decreased gastrointestinal motility [7–10].

In hospitalized patients, a continuous infusion of ketamine, an anesthetic drug which is also an NMDA antagonist, is commonly administered with traditional analgesics for managing wind-up pain [11]. Although ketamine is widely used for its NMDA receptor antagonist properties, the safety and efficacy of other drugs reported to produce NMDA antagonist effects have not been investigated for acute or chronic equine pain management. Amantadine, which was historically been used to prevent influenza A is an NMDA receptor antagonist drug that has a multitude of uses including treatment of Parkinson’s disease in humans [12] and osteoarthritis pain in small animals and people [13, 14, 12]. In humans and dogs, amantadine has shown efficacy when used in combination with opioids and NSAIDs, for the treatment of musculoskeletal conditions associated with central sensitization [13, 15, 16].

The potential to use amantadine in managing maladaptive pain in horses is limited by a lack of data investigating the pharmacological safety, pharmacokinetic parameters, and therapeutic dosage regimens. While there are no published studies describing analgesic efficacy in horses, there is a single study describing the pharmacokinetics of this drug in this species [17]. In that study, amantadine was variably absorbed following oral administration of 10 and 20 mg/kg in horses (40–60%) [17]. The high variability between animals along with the limited number of horses studied, supports further investigation to ensure adequate dosing for assessment of pharmacologic effects. In the previous study in horses, while oral administration of amantadine appeared was well-tolerated, IV administration of a 20 mg/kg dose resulted in transient neurological effects, including acute seizures and inconsistent limb placement [17]. These findings warrant further investigation of the safety of amantadine administration in horses. The goal of the current study was to describe the plasma concentrations and pharmacokinetic parameters of amantadine and to describe physiological and behavioral effects following oral and IV administration to horses.

## Methods

### Animals

Eight healthy, university-owned, Thoroughbred horses, 5 geldings and 3 mares, aged 3–6 years, and weighing 547.6 ± 39.2 kg [mean ± standard deviation] were studied. The horses were determined to be healthy by physical examination, a normal complete blood count, and a serum biochemistry panel. The complete blood count and serum biochemistry analyses were performed by the Clinical Pathology Laboratory of the William R. Pritchard Veterinary Medical Teaching Hospital of the University of California, Davis, using standard protocols. Horses did not receive any medications for a minimum of two weeks before beginning the study. Food was withheld for 10–12 h before drug administration and for 2 h post administration. The study was conducted in accordance with the Institutional Animal Care and Use Committee of the University of California, Davis (Protocol #24336).

### Study design and drug administration

This study was conducted in a balanced, randomized four-way cross-over design. Horses were randomized with respect to the order of treatment with a 2-week washout period observed between each amantadine dose for each horse. A single intravenous (IV) administration of 5 mg/kg and oral administrations of 3, 5, and 10 mg/kg of amantadine HCl (Alembic Pharmaceuticals Limited, Bridgewater, NJ) were administered to each horse. The doses of 3 and 5 mg/kg were selected based on suggested efficacious doses in other species (dogs, cats, birds, and humans) [1–4, 13, 14, 18, 19]. A dose of 10 mg/kg was selected since oral bioavailability is often lower in horses compared to other species, and to allow for assessment of non-linearity with respect to absorption and/or elimination. An oral dose of 20 mg/kg was well tolerated in a previous study in horses that assessed the use of amantadine as an anti-viral agent [17]. As IV amantadine was administered only to determine bioavailability, a low dose of 5 mg/kg was selected. An IV dose of 10 mg/kg was well tolerated in a previous study in horses [17].

Since an IV formulation is not commercially available, the IV formulation was compounded from a pharmaceutical-grade amantadine HCL powder (purity *≥* 99%; Sigma Aldrich Inc, St Louis, MO) using sterile technique. The powder was dissolved in sterile water and filter sterilized prior to administration. The drug was administered within 1-hour of formulation. An aliquot of the dosing formulation was saved, and the concentration was confirmed by liquid chromatography-tandem mass spectrometry (LC-MS/MS). The IV formulation was administered as an IV bolus via a jugular vein catheter placed immediately prior to drug administration. The administration catheter was removed following dosing. Amantadine oral tablets were suspended in water immediately prior to administration and given through a dosing syringe directly into the oral cavity. The dose was administered slowly, and swallowing was confirmed before the horse’s head was released, to maximize the likelihood that the full dose was administered.

### Blood sample collection

For sample collection, a 14-gauge catheter was placed in one external jugular vein (opposite of the dosing catheter for IV administration). Blood samples were collected at time 0 (before drug administration) and at 5, 10, 15, 30, and 45 min, as well as 1, 2, 3, 4, 5, 6, 8, 12, and 18 h following administration. The catheters were removed after the 18-hour sample collection, and the remaining samples were collected at 24, 30, 36, 48, 72, and 96 h following administration by direct venipuncture. Blood samples were collected in EDTA tubes and placed on ice until centrifugation at 3000 x g. After centrifugation, the plasma was immediately transferred into storage cryovials and stored at -20 °C until analyzed for drug concentrations.

### Determination of amantadine concentrations

Amantadine concentrations were quantified using an LC–MS/MS method adapted from a previously published and validated assay [20] A partial validation was conducted to confirm assay performance under the conditions of the present study. Accuracy and precision were assessed through analysis of quality control samples included with each analytical run and met predefined acceptance criteria for accuracy (concentration within ± 15% of nominal concentrations) and precision(≤ 15% relative standard deviation).

Amantadine plasma calibrators were prepared by dilution of the amantadine working standard solutions (Sigma Aldrich Inc, St. Louis, MO) with drug free equine plasma to concentrations ranging from 0.1 to 7,500 ng/mL. Calibration curves and negative control samples were prepared fresh for each quantitative assay.

For sample extraction, plasma (0.5 mL) was diluted with 0.5 mL of acetonitrile (ACN):1 M acetic acid (9:1, v: v) containing 0.1 ng/mL of the internal standard (memantine, Sigma Aldrich, St Louis, MO), to precipitate proteins. Plasma samples were subsequently vortexed for 1 min to mix, refrigerated for 20 min, vortexed for an additional 1 min, and centrifuged (4300 rpm/3102 g) for 10 min at 4 °C prior to injection of 10 µL into the LC-MS/MS system.

Concentrations of the analytes were measured in plasma by LC-MS/MS using positive heated electrospray ionization (HESI(+)). A TSQ Altis triple quadrupole mass spectrometer coupled with a Vanquish liquid chromatography system (Thermo Scientific, San Jose, CA) was used for quantitative analysis. Chromatography employed an ACE 3 C18 10 cm x 2.1 mm 3 μm column (Mac-Mod Analytical, Chadds Ford, PA) and a linear gradient of ACN in water with a constant 0.2% formic acid at a flow rate of 0.35 ml/min. The initial ACN concentration was held at 1% for 0.3 min, ramped to 90% over 4.8 min and held at that concentration for 0.1 min, before re-equilibrating for 2.9 min at initial conditions.

Selective Reaction Monitoring (SRM) of initial precursor ion for amantadine (mass to charge ratio *(m/z)* 152.1) and memantine (*(m/z)* 180.1) was used for detection and quantification. The response for the product ions for amantadine (*m/z* 135.1) and memantine (*m/z* 107.0, 163.0) were plotted, and peaks at the proper retention time integrated, using Quanbrowser software (Thermo Scientific). Quanbrowser software was used to generate calibration curves and quantitate amantadine in all samples by linear regression. A weighting factor of 1/X was used for all calibration curves.

### Pharmacokinetic analysis

Noncompartmental analysis (NCA) was performed on amantadine plasma concentrations using Phoenix WinNonlin software (V8.3; Certara, Princeton, NJ). Concentrations less than the limit of quantitation (LOQ) of the assay were excluded from the pharmacokinetic analysis. The maximum concentration (C_max_) and time of maximum concentration (T_max_) were determined from the concentration data. The C_max_ and area under the curve extrapolated to infinity (AUC_inf_) are reported as absolute as well as dose normalized values (C_max_/D and AUC_last_/D) for comparison between dose groups. The AUC_inf_ was determined using the log up-linear down trapezoidal method.

The oral bioavailability (F) for each horse at each dose was calculated using the formula:

$$\:F=\left(\raisebox{1ex}{${AUC}_{oral}$}\!\left/\:\!\raisebox{-1ex}{${AUC}_{IV}$}\right.\right)*\:\left(\raisebox{1ex}{${Dose}_{IV}$}\!\left/\:\!\raisebox{-1ex}{${Dose}_{oral}$}\right.\right)$$

Where AUC_oral_ represents AUC_inf_ following oral administration and AUC_IV_ is AUC_inf_ following IV administration.

Total systemic clearance (Cl) was calculated using the formula:

$$\:Cl=\raisebox{1ex}{${Dose}_{IV}$}\!\left/\:\!\raisebox{-1ex}{${AUC}_{inf}$}\right.$$

For the oral doses, derived clearance was calculated for individual horses at each dose for comparison of linearity with respect to clearance between doses.

$$\:{Cl}_{derived}={AUC}_{inf\:}x\:F$$

Where AUC_inf_ represents the AUC following oral administration and F represents oral bioavailability.

Following NCA, concentration data for all horses from both routes of administration (IV and oral) were modeled simultaneously using a nonlinear mixed effect (NLME) population modeling approach with the Phoenix software. Only concentrations greater than or equal to the LOQ were included in the analysis. The first-order conditional estimation method with interaction was used in the model-building process. Both 2 and 3-compartment models were evaluated. Residual error models including additive, multiplicative, and Poisson were all considered. Random effects were included for all structural variables and were modeled with log linear functions. A log-normal distribution was used for all pharmacokinetic parameters except bioavailability, which was modeled using a logit-normal distribution because it is constrained between 0 and 1. For the random effects a diagonal variance-covariance matrix was used. Visual analysis of the observed versus predicted concentration graphs, residual plots, Akaike Information Criterion, %CV, and − 2LL were all considered in assessing which model provided the best fit.

### Physiological and behavioral assessments

Horses were equipped with two Step Monitors (SAM3, Seattle, WA) to count the number of steps taken each minute. The step counters were fastened with a Velcro strap on the lateral aspect of the distal left thoracic limb and the distal right pelvic limb. Straps without a monitor were placed at the distal aspect of the other two limbs to minimize the horses favoring a particular limb. The monitors recorded the number of steps taken for 10 min before and 8 h after drug administration. The number of steps for individual horses was summed in 10-minute increments and the average ± SD for all horses reported for each dose.

A Holter monitor (Forrest Medical, East Syracuse, NY) was placed on each horse for continuous recording of heart rate and rhythm for 30 min before and 8 h after drug administration. Heart rate was calculated by manual counting of P-QRS-T complexes over the course of one minute.

Gastrointestinal borborygmi were determined to be normal, increased, decreased, or absent following auscultation in each of the four quadrants before and at 30 and 45 min, as well as 1, 2, 4, 6, 8, and 24 h following administration. Auscultation was performed for 15 s at each quadrant, and a numerical score representative of the number of borborygmi per 30 s in each quadrant was determined. Auscultation was performed following collection of blood samples by an individual not blinded to treatment. Changes in fecal consistency and defecation incidence were also recorded during the sampling period.

Subjective assessments of disposition (i.e. sedation, excitation, etc.) were conducted by observers familiar with normal behaviors in these horses. Observations were recorded before entering each horse’s stall to collect blood samples. A modified neurological exam, primarily to assess the degree of ataxia, was performed in horses exhibiting neurological signs post drug administration. Ataxia was graded using the Mayhew grading system (scale from 0–5) [21].

### Statistical analysis

Statistical analyses were conducted using commercially available software (Stata/BE 17.0 BE, StataCorp, College Station, TX, USA) to assess significant differences in pharmacodynamic parameters between baseline and each time point within each dose group. All analyses were performed with mixed effects analysis of variance, in which the horse is the random effect, and time is the fixed effect. Post hoc comparisons were performed with a Bonferroni multiple comparison adjustment to preserve a nominal significance level of *p* < 0.05.

Differences in pharmacokinetic parameters among treatment groups were evaluated using linear mixed-effects models with treatment included as a fixed effect and horse included as a random effect. Pairwise comparisons were performed using Bonferroni-adjusted post hoc tests. The AUC_inf_ and λz were analyzed on the log-transformed scale, whereas AUC_inf_/D, C_max_, C_max_/D, CL/F, t½, F, and T_max_ were analyzed on their original scales. Statistical significance was set at *p* < 0.05. T_max_ was summarized descriptively as median (range), while all other pharmacokinetic parameters were summarized as mean ± SD. Statistical significance was set at *p* < 0.05.

## Results

The response for amantadine was linear and gave correlation coefficients of 0.99 or better. Quality control samples were assayed in replicates (*n* = 6) for determination of precision and accuracy. Accuracy was reported as percent nominal concentration and precision as percent relative standard deviation (Supplementary Table 1). The technique was optimized to provide a LOQ of 0.1 ng/mL and a limit of detection (LOD) of approximately 0.05 ng/mL for amantadine.

**Fig. 1:**
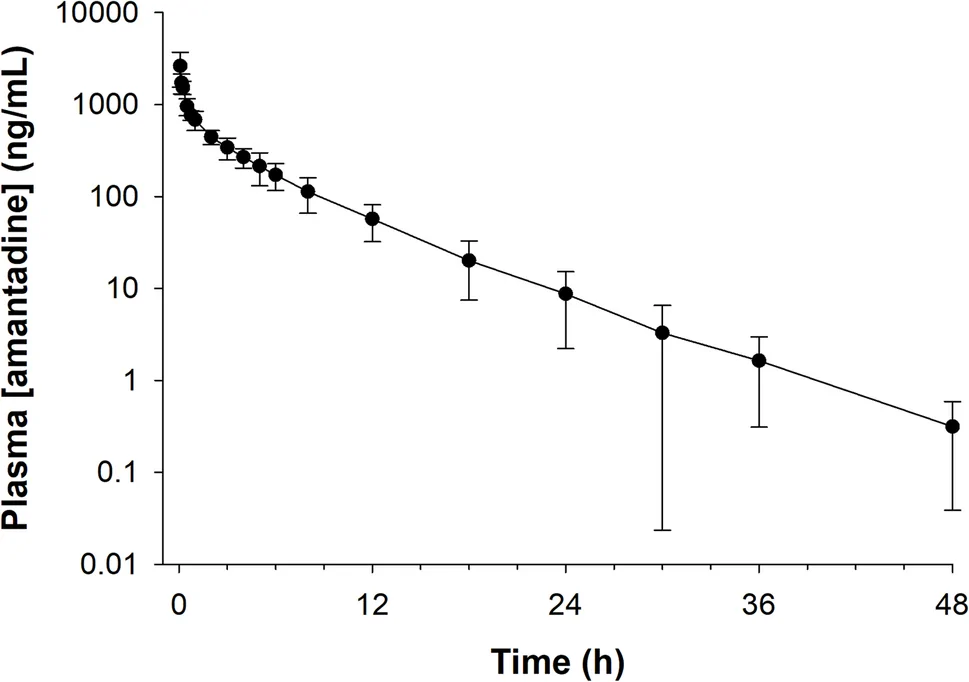
Mean ± standard deviation plasma amantadine concentrations following a single IV administration of 5 mg/kg to 8 horses

**Fig. 2:**
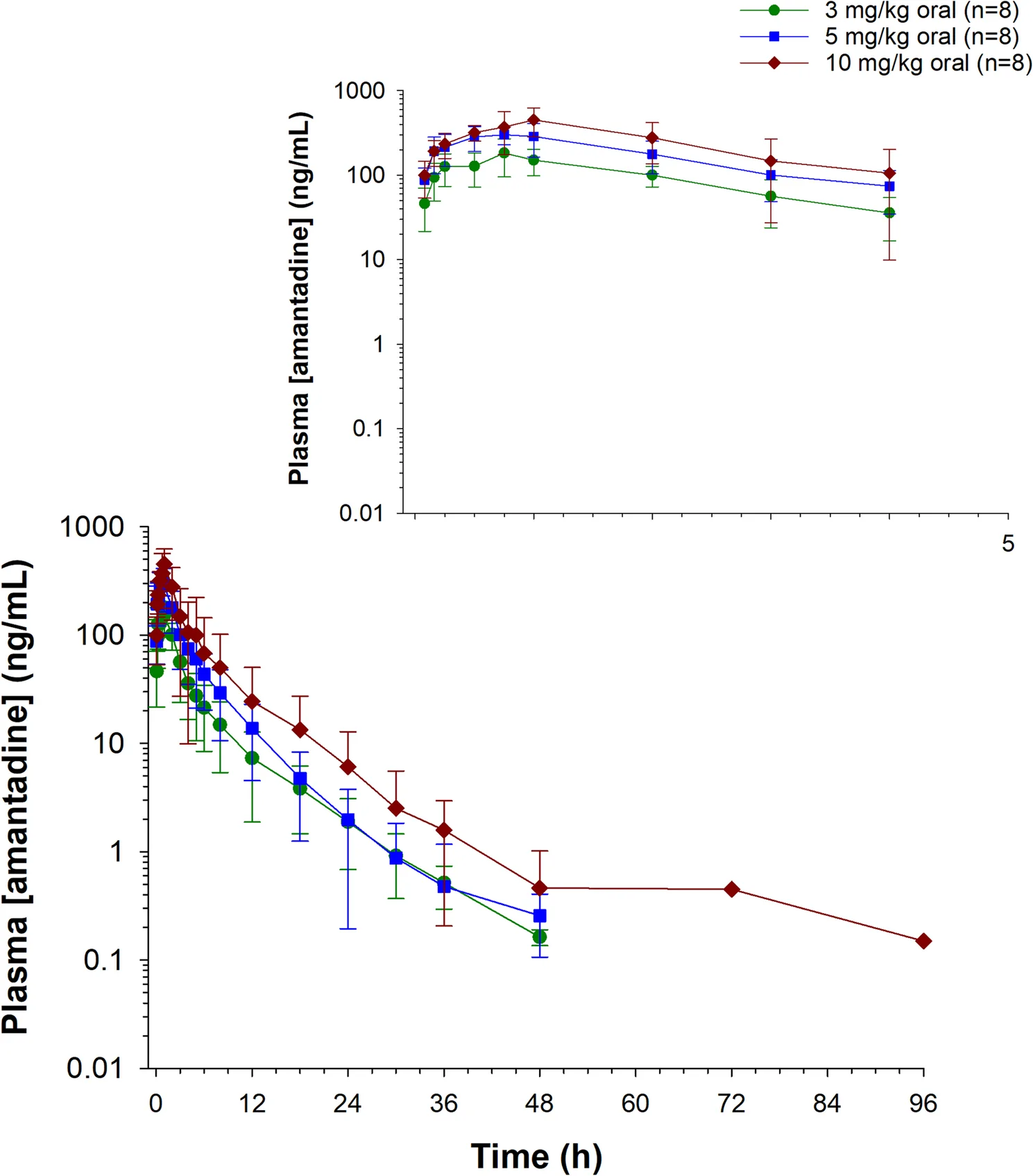
Mean ± standard deviation plasma amantadine concentrations following a single oral administration of 3, 5, and 10 mg/kg to 8 horses. The inset shows the first 4 h

Plasma amantadine concentrations after a single IV administration of 5 mg/kg and oral administration of 3, 5, and 10 mg/kg are shown in Figs. 1 and 2. Plasma amantadine concentrations were above the LOQ in 5/8 horses at 48 h post IV administration and decreased below the LOQ (0.1 ng/mL) of the assay by 72 h in all horses. Drug concentrations were below the LOQ by 72 h following oral administration of 3 and 5 mg/kg. For the 10 mg/kg oral dosing group, the plasma amantadine concentrations decreased below the LOQ of the assay by 72 h after administration in all but one horse.

Pharmacokinetic parameters (mean ± SD), generated from NCA, for all dosing groups are listed in Table 1. Time to maximum plasma concentrations was longer for the 10 mg/kg PO dose (T_max_ = 1 h) and similar between the 3 mg/kg and 5 mg/kg PO doses (T_max_ = 0.63 h). There was not a significant difference in the dose normalized C_max_ or AUC_inf_ (C_max_/D, AUC_inf_/D) or bioavailability between oral doses. The %AUC_extrap_ was ≤ 20% for each dosing group. Clearance, corrected for oral bioavailability was similar to IV and between oral doses. The terminal half-life was also similar between both routes and all doses.

**Table 1:** Pharmacokinetic parameters for amantadine following a single IV (5 mg/kg) and oral (3, 5, or 10 mg/kg) administration to 8 horses. Parameters generated using non-compartmental analysis

Parameter	Intravenous	Oral		
	5 mg/kg	3 mg/kg	5 mg/kg	10 mg/kg
C(0) (ng/mL)	4,257.6 ± 3087.4	---	---	---
C_max_ (ng/mL)	---	208.7 ± 70.4^bc^	347.9 ± 79.5^ac^	478.9 ± 165.6^ab^
C_max_/D (ng/mL)	---	69.8 ± 21.9	69.6 ± 15.9	47.9 ± 16.6
T_max_ (h)	---	0.63 (0.16–0.75)^c^	0.63 (0.16-1.0)^c^	1.0 (0.5-1.0)^ab^
AUC_inf_ (h*ng/mL)	3,921.2 ± 917.2	554.8 ± 193.9^bc^	1,042.9 ± 393.4^a^	1,614.8 ± 1,123.4^a^
AUC_inf_ /D (h*ng/mL)	---	184.6 ± 65.0	208.7 ± 78.8	160.3 ± 109.9
AUC % extrap	0.057 ± 0.050	0.236 ± 0.125	0.238 ± 0.167	0.513 ± 1.06
Bioavailability (%)	---	24.7 ± 9.88	27.2 ± 11.3	20.2 ± 8.65
Cl/F (mL/min/kg)	---	6,508.5 ± 3,812.9	5,854.9 ± 3,639.3	8,400.7 ± 4,163. 0
Cl (mL/min/kg)^*^	22.2 ± 4.64	21.4 ± 4.45	22.2 ± 4.61	21.4 ± 4.38
Vd_ss_ (L/kg)	5.47 ± 1.32	---	---	---
Lambda z(1/h)	0.195 ± 0.064	0.254 ± 0.168	0.181 ± 0.058	0.179 ± 0.104
Terminal t_1/2_ (h)	3.90 ± 1.24	3.87 ± 2.17	4.21 ± 1.41	4.87 ± 2.05
MRT_inf_ (h)	4.20 ± 1.02	---	---	---

C(0), concentration in the plasma at time 0 following IV administration; C_max_, maximum plasma drug concentration; C_max_/D, maximum plasma drug concentration corrected for dose; T_max_, time of maximum plasma drug concentration; AUC_inf,_ area under the curve extrapolated to infinity; AUC_inf_ /D, area under the curve extrapolated to infinity corrected for dose, AUC % extrap, percent of area under the curve extrapolated; Cl/F, apparent clearance; Cl, total clearance; Vd_ss_, volume of distribution at steady state; Lambda z, terminal slope, MRT_inf_, mean residence time extrapolated to infinity

^*^, for oral administration Cl was calculated for individual horses (before taking the mean) using the formula: Cl/F x F

^a,^ indicates a significant difference (*p* < 0.05) compared to the 3.0 mg/kg oral dose group; ^b^, indicates a significant difference (*p* < 0.05) compared to the 5 mg/kg oral dose group; ^c^, indicates a significant difference (*p* < 0.05) compared to the 10 mg/kg oral dose group

Using an NLME population PK model compartmental modeling, based on evaluation of goodness of fit criteria, a 2-compartment multiplicative error model, parameterized with respect to clearance, gave the best fit to the data. The diagnostic plots used to assess the fit are provided are provided as Supplementary Figures. Pharmacokinetic parameters (estimate and % coefficient of variation for the fixed and random effects) are listed in Table 2.

**Table 2:** Model typical values (tv) for amantadine following a single IV (5 mg/kg) and oral (3, 5, or 10 mg/kg) administration to 8 horses

	Fitted Model Parameters	CV(%)	Eta Shrinkage (%)
Fitted Model Parameters			
tvKa (1/h)	3.16	20.3	10.9
tvV (L/kg)	3.19	11.8	8.75
tvV2 (L/kg)	2.17	10.7	23.2
tvCl (mL/min/kg)	22.4	7.92	6.54
tvCl2 (mL/min/kg)	6.78	18.4	16.5
tvFbio (%)	23.2	13.6	2.83
Derived Parameters			
K10 HL (h)	1.64	13.6	
Effective HL (h)	2.76	10.2	
Vd_ss_ (L/kg)	5.36	8.16	
stdev0	0.40	3.82	
Between subject variability (%CV)			
V	0.093	31.1	
V2	0.039	19.9	
Cl	0.042	20.6	
Cl2	0.154	40.8	
Fbio	0.138	38.5	

tvKa, rate of absorption; tvV, the value of the central compartment; tvV2, denotes the value of the peripheral compartment; tvCl, the clearance of drug from plasma; tvCL2, the clearance of drug from the peripheral compartment; tvlag, lag time; tvFbio = bioavailability; K10 HL = K10 half-life. Alpha HL, half-life of distribution; Beta HL, beta half-life; Vd_ss_, volume of distribution at steady state; stdev0 = the estimated residual standard deviation for plasma data. CV(%), coefficient of variation

Mild sedation (standing quietly, facing wall with head lowered, eyes closed) was observed in 2/8 horses 1 h after administration of 3 mg/kg and 5 mg/kg PO. Mild neurological signs were exhibited by horses in the 5 mg/kg IV dose group from 5 to 15 min post administration. Three of 8 horses displayed Grade I ataxia at 5 and 15 min post IV administration. 2/8 horses displayed Grade II ataxia at 5 and 10 min post IV administration. Hypometria was seen in 1/8 horses at 5 min after IV administration.

**Fig. 3:**
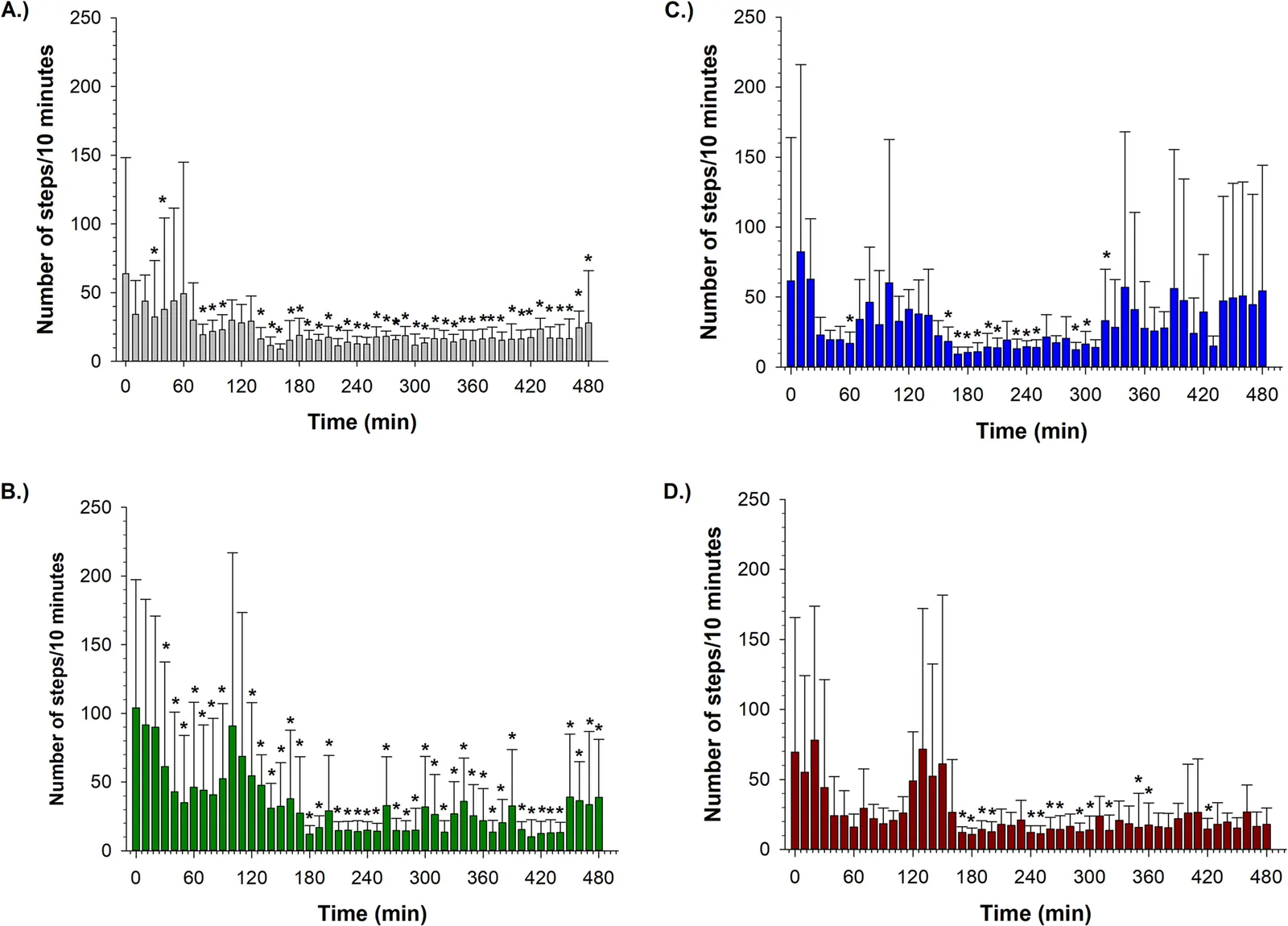
Number of steps per 10 min (mean ± SD) following administration of amantadine (**A**) 5 mg/kg IV; *n* = 8 horses, (**B**) 3 mg/kg oral; *n* = 8 horses, and (**C**) 5 mg/kg oral; *n* = 8 horses and (**D**) 10 mg/kg; *n* = 8 horses. * Indicates a statistically significant (*p* < 0.05) change from baseline

Step counts at baseline to 480 min post administration for all dosing groups are shown in Fig. 3. Step counts were significantly decreased relative to baseline for the 3 mg/kg oral dose group from 30 to 90 and 120 to 480 min post administration. Step counts were significantly decreased compared to baseline for the 5 mg/kg oral dose group at 60, 160 to 210, 230 to 250, and 290 to 310 min post administration. For the 10 mg/kg oral dose group, step counts were significantly decreased relative to baseline at 170 to 200, 240 to 270, 290 to 300, 320, 350 to 360, 380, and 420 min after administration. For the 5 mg/kg IV dose group, step counts were also significantly decreased relative to baseline at 30 to 40, 80 to 100, and 140 to 480 min post administration.

Heart rate was significantly decreased relative to baseline at 8, 15, and 20 min and 1, 1.25, 1.5, and 2 h post administration in the 3 mg/kg oral dose group (Table 3). Heart rate was significantly increased at 4.5 to 5 and 6 to 6.5 h after administration in the 5 mg/kg oral dose group, and at 2 min and 2.5 to 8 h in the 10 mg/kg oral dose group (Table 3). Heart rates at each time point and across dose groups were all within the clinically normal range.

**Table 3:** Heart rate (mean ± SD beats minute), following a single IV (5 mg/kg) and oral (3, 5, 10 mg/kg) administration to 8 horses

Time (h)	3 mg/kg PO	5 mg/kg PO	10 mg/kg PO	5 mg/kg IV
0	39 ± 7	35 ± 8	34 ± 6	39 ± 8
0.03	35 ± 8	38 ± 7	39 ± 7*	38 ± 8
0.08	35 ± 7	35 ± 5	36 ± 5	40 ± 8
0.13	34 ± 9*	32 ± 5	38 ± 10	41 ± 10
0.17	36 ± 17	32 ± 5	35 ± 5	40 ± 9
0.20	35 ±14	31 ± 4	35 ± 6	37 ± 6
0.25	33 ± 12*	32 ± 3	34 ± 6	37 ± 6
0.33	33 ± 8*	36 ± 10	34 ± 7	38 ± 5
0.5	36 ± 9	35 ± 8	34 ± 6	38 ± 7
0.75	35 ± 8	36 ± 11	32 ± 6	40 ± 7
1	34 ± 7*	35 ± 9	33 ± 6	39 ± 6
1.25	33 ± 6*	32 ± 9	32 ± 6	39 ± 9
1.5	32 ± 7*	33 ± 7	33± 5	37 ± 7
2	30 ± 5*	32 ± 4	32 ± 6	35 ± 5
2.5	37 ± 4	39 ± 7	38 ± 6*	40 ± 5
3	39 ± 5	39 ± 6	39 ± 4*	41 ± 5
3.5	43 ± 9	39 ± 4	40 ± 6*	40 ± 5
4	38 ± 3	37 ± 6	39 ± 6*	40 ± 6
4.5	39 ± 6	40 ± 6*	39 ± 5*	41 ± 5
5	38 ±5	42 ± 6*	43 ± 6*	39 ± 5
5.5	43 ± 4	39 ± 4	39 ± 6*	40 ± 6
6	36 ± 4	41 ± 4*	39 ± 5*	39 ± 4
6.5	39 ± 5	41 ± 6*	40 ± 4*	39 ± 6
7	36 ± 3	41 ± 4	39 ± 3*	38 ± 5
7.5	40 ± 3	38 ± 3	40 ± 5*	40 ± 4
8	43 ± 5	38 ± 4	38 ± 3*	39 ± 3

*indicates a significant difference (*p* < 0.05) relative to baseline of each dosing group

Gastrointestinal borborygmi scores are listed in Table 4. Gastrointestinal sounds were significantly increased relative to baseline at 4 to 6 h after administration for the 3 mg/kg oral dose group and at 6 h post administration for the 5 mg/kg oral dose group. In the 5 mg/kg IV dose group, GI sounds were significantly increased relative to baseline at 4 to 24 h post administration. GI sounds were not significantly different relative to baseline for the 10 mg/kg oral dose group. Subjectively, the GI borborygmi scores were comparable between groups.

**Table 4:** Gastrointestinal scores (mean ± SD), following a single IV (5 mg/kg) and oral (3, 5, 10 mg/kg) administration to 8 horses

Time (h)	3 mg/kg PO	5 mg/kg PO	10 mg/kg PO	5 mg/kg IV
Baseline	2.9 ± 0.8	2.4 ± 1.5	2.4 ± 0.9	1.8 ± 0.8
0.5	1.8 ± 1.3	2.8 ± 1.4	2.8 ± 1.2	2.0 ± 1.2
0.75	2.1 ± 1.2	2.8 ± 1.2	3.0 ± 1.1	2.4 ± 0.9
1	2.5 ± 0.9	2.9 ± 1.2	2.8 ± 1.5	1.8 ± 1.2
2	2.4 ± 1.0	2.5 ± 1.1	3.1 ± 0.8	2.3 ± 1.2
4	3.6 ± 0.9*	4.0 ± 0.9	2.9 ± 0.3	2.9 ± 1.4*
6	3.0 ± 0.7*	3.9 ± 0.9*	3.5 ± 0.7	3.5 ± 1.0*
8	3.4 ± 1.1	3.3 ± 1.1	2.9 ± 1.2	3.4 ± 1.1*
24	3.0 ± 1.1	3.3 ± 0.8	3.0 ± 0.9	2.8 ± 0.8*

*indicates a significant difference (*p* < 0.05) relative to baseline of each dosing group

## Discussion

The current study describes the pharmacokinetics of a single IV dose and oral administration of three doses of amantadine in horses. It is important to note that while the administration of amantadine in the United States at the current time would be considered extra-label drug use, in the European Union amantadine is not authorized for veterinary use [22]. Intravenous administration was included for the purpose of determining bioavailability. Both non-compartmental and population compartmental analysis were conducted. As reported previously for amantadine in horses [17] and cats [23], in the current study, a 2-compartment model best fit the plasma concentration.

In the presently reported study, C_max_ increased proportionally with increasing doses, suggesting linear absorption. Further supporting linear absorption kinetics across the doses studied was the dose proportionate increase in AUC and similar oral bioavailability for all doses.

Oral bioavailability was low to moderate (24.7 ± 9.88, 27.2 ± 11.3, and 20.2 ± 8.65% for 3, 5, and 10 mg/kg, respectively). A previous investigation of the pharmacokinetics of amantadine in horses reported an oral bioavailability of 40% − 60% in the fed state and 39% when food was withheld [17]. This disparity between the studies may be a result of differences in the methods of administration. In the current study, the drug was administered via a dosing syringe, directly into the oral cavity, to mimic a clinical scenario. In the study conducted by Rees and colleagues amantadine was delivered via a nasogastric tube [17]. Although every attempt was made to ensure horses received the full dose there is always a possibility of loss of drug when administering drug via a dosing syringe into the oral cavity. This offers a possible explanation for the difference between the current and the previous study. Rees et al. also reported a high degree of variability in oral bioavailability [17]. In the current study, the inter-subject variability, determined from population modeling, was also high (38.5%). Overall, oral bioavailability is lower in horses compared to cats (130 ± 11%) [23], dogs (86–90%) [24], and mice (58.6%) [24]. This is not surprising as oral absorption of many drugs is less complete in horses, compared to other monogastric species [25, 26].

Systemic clearance of amantadine (22.4 ± 4.64 mL/min/kg) in this study was lower than that reported by Rees et al. (36.8 ± 12.4 mL/min/kg) [17]. The most likely explanation for the discrepancy is differences in the sampling protocol and/or differences in the sensitivity of the analytical assays used to determine amantadine concentrations. The LOQ in the current study was 0.1 ng/mL, whereas in the previous study, the limit of sensitivity was 50 ng/mL [17]. In the previous study, samples were collected and concentrations fell below 50 ng/mL by 8 h post drug administration [17]. In the current study, amantadine concentrations were still above the LOQ (0.1 ng/mL) at 48 h post administration in 3/8 horses, falling below the LOQ by 72 h in all horses. The inclusion of additional time points allows for more precise calculation of the elimination rate and a more complete calculation of the AUC, reducing potential extrapolation error. This likely also explains the differences in the reported terminal half-life between the current (3.90 ± 1.24 h) and previous equine study (1.83 ± 0.87 h) [17] following IV administration. While the terminal half-life following IV administration differed between the studies, the half-life following oral administration was similar. Additionally, in the current study, the terminal half-life was comparable between IV (3.90 ± 1.24 h) and oral doses (3.87 ± 2.17, 4.21 ± 1.41, and 4.87 ± 2.05 h for 3, 5 and 10 mg/kg, respectively) and is similar to what has been reported for cats (5.8 h) [23] and dogs (5.9 h) [18] but shorter than for humans (9–16 h) [27–30]. Comparable half-lives between oral doses supports linear elimination kinetics for amantadine, across the studied dose range. As an additional check of linearity, derived clearance (Cl corrected for bioavailability) following oral administration was also calculated and compared to systemic clearance following IV administration. The derived clearance was consistent across the oral doses studied and agreed with IV clearance, further supporting linear clearance.

The Vd_ss_ in the current study (5.47 ± 1.32 L/kg) agreed with the previous report in horses (4.87 ± 1.9 L/kg) [17]. This is also similar to what has been reported for cats (4.3 ± 0.2 L/kg) [23]. In dogs, the Vd is reportedly much larger at 13.8 L/kg [24].

Following NCA, plasma concentration data was modeled using a population model, allowing for the determination of between-subject variability. Another advantage to population modeling of pharmacokinetic data is that is allows for the assessment of the effects of known covariates (age, weight, gender, etc.) on specific pharmacokinetic parameters. As discussed previously, the between subject variability for bioavailability was high. Additionally, the inter-compartmental clearance (Cl2) was also highly variable between horses (40.8%). Unfortunately, the small sample size in the current study precluded assessment of covariate effects, but the population model described here does provide a base model that can be used to understand covariate effects in the future as additional pharmacokinetic studies are conducted and additional horses are added to the model.

To the best of the authors’ knowledge, there are no published reports describing analgesic concentrations of amantadine in any species. In dogs, an oral dosage of 3–5 mg/kg q24 hours is recommended. In one study, the C_max_ following a dose of 3 mg/kg ranged from 225 to 351 ng/mL with 12- and 24-hour concentrations reported as 48.5 and 13.4 ng/mL (geometric mean), respectively [18]. While notably therapeutic concentrations may vary between species, in the current study, plasma amantadine concentrations following administration of an oral dose of 10 mg/kg resulted in a C_max_ of 478.9 ng/mL and a 12-hour concentration of 18.1 ng/mL. These findings suggest that a dose of 10 mg/kg may achieve concentrations equivalent to those achieved following administration of a dose considered to be clinically effective in dogs (5 mg/kg) [18].

Significant decreases in locomotion (the number of steps taken) were noted at various times post drug administration in all treatment groups, with the IV group seemingly the most affected. Transient (up to 15 min post administration) neurological signs, including ataxia and hypometria were observed in 5/8 horses following IV administration (5 mg/kg). Similarly, in a previous study, investigators reported CNS effects including seizures (20 mg/kg IV), and other transient (lasting 30–60 min) CNS signs including stumbling, inconsistent limb placement, dragging of toes, weakness in lower back muscles following IV administration of 15 mg/kg [17]. The investigators did not study the specific plasma amantadine concentration that precipitated the seizures, but they did report that most likely concentrations would have been greater than 2000–4000 ng/mL [17]. Although seizures were not noted in the current study, in horses that showed neurologic effects, plasma concentrations ranged from 2,000 to 4,000 ng/mL (2,365.9–4,743.2 ng/mL in 5/8 horses at 5 min and 2,215.3-2,348.4 ng/mL in 2 horses at 10 min) at the time these signs were observed. Neurologic effects were not observed in any of the horses receiving oral amantadine, likely because plasma amantadine concentrations were lower compared to the IV group.

Alterations in gastrointestinal motility is a common undesirable effect following administration of commonly used analgesic medications, such as NSAIDs and opioids, in horses [10, 31–34]. In the current study, effects on gastrointestinal borborygmi were limited, with increases observed at later times points post administration, primarily in the IV treatment group. While this is favorable with respect to the gastrointestinal safety profile of amantadine in horses, it is important to consider that assessment of gastrointestinal borborygmi by direct auscultation is a subjective measure of gastrointestinal motility and is subject to observer variability.

An important limitation of the current study is that it was performed in healthy horses. It is important to note that pharmacokinetics may differ in horses with clinical pain or systemic illness, compared to healthy horses. Future studies should include characterization of the pharmacokinetics of amantadine in horses with both acute and chronic clinical pain.

## Conclusion

Current options for pain management in horses, especially chronic and neuropathic pain, are limited. In the current study in horses, plasma concentrations and pharmacokinetics of amantadine, an adjunct medication used in combination with NSAIDs or opioids for the management of chronic or neuropathic pain in people and other animal species, are described. Oral administration of amantadine to horses was well tolerated, with a bioavailability of 23.2% and a systemic clearance of 22.4 mL/min/kg. Plasma amantadine concentrations achieved in the current study, support oral administration of 10 mg/kg every 12–24 h as a starting dose for pharmacodynamic efficacy studies in horses.

## Supplementary Information

Supplementary Material 1.

Supplementary Material 2.

## Data Availability

All data supporting the findings of this study are available within the paper and its Supplementary Information.

## Abbreviations

ACN: Acetonitrile
AUC_inf_: Area under the curve extrapolated to infinity
Cl: Clearance
C_max_: Maximum concentration
F: Bioavailability
IV: Intravenous
LC-MS/MS: Liquid chromatography-tandem mass spectrometry
LOD: Limit of detection
LOQ: Limit of quantitation
NCA: Noncompartmental analysis
NLME: Nonlinear mixed effect
NMDA: N-methyl-D-aspartate
NSAID: Non-steroidal anti-inflammatory drugs
T_max_: Time of maximum concentration
