# Supplementary material for: Assessment of pharmacokinetic parameters, oral bioavailability, and physiological effects of amantadine in horses
Source: BMC Vet Res. 2026 Jun 23;22:498. doi: 10.1186/s12917-026-05672-9 (PMC13520257; doi:10.1186/s12917-026-05672-9)
Supplement: Supplementary file 1 [file 12917_2026_5672_MOESM2_ESM.pdf]

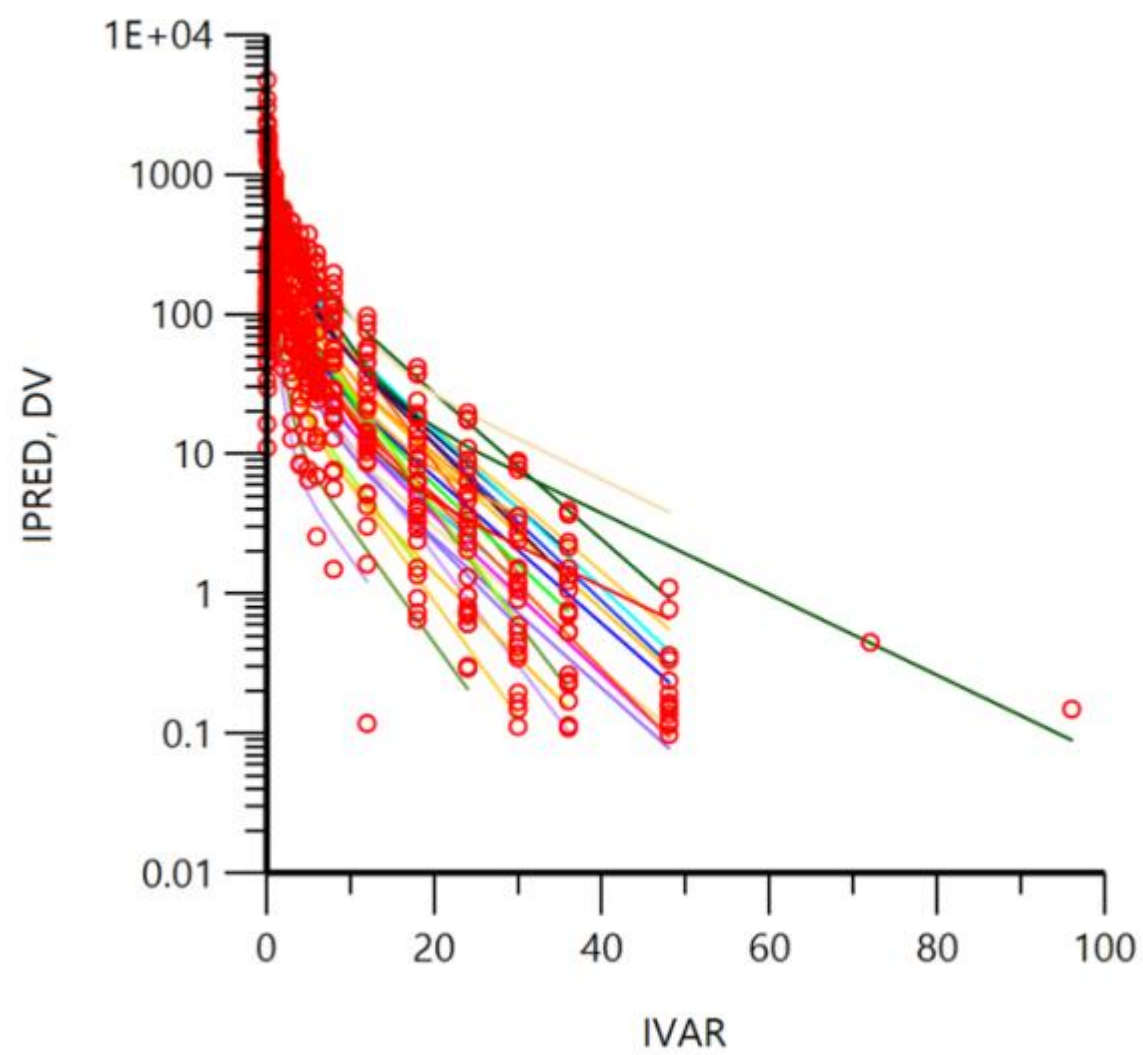

Supplementary Figure 1. Plot of dependent variable (DV; concentrations) and individual prediction (IPRED) vs independent variable (IVAR; time)**.**

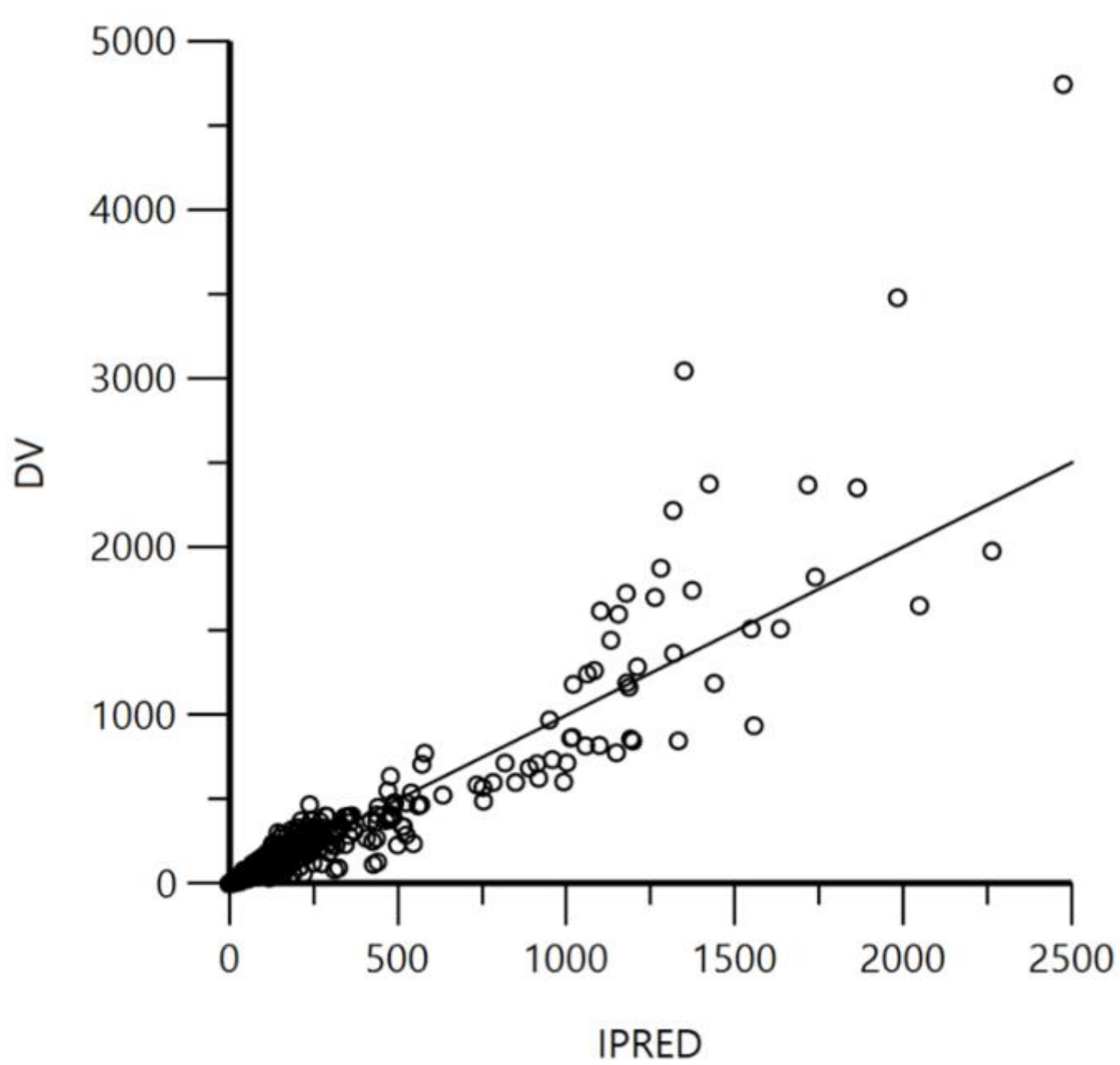

Supplementary Figure 2. (A) Plot of the dependent variable (DV; concentrations) versus individual predicted values (IPRED; predicted concentrations).
