# Supplementary material for: Assessment of pharmacokinetic parameters, oral bioavailability, and physiological effects of amantadine in horses
Source: BMC Vet Res. 2026 Jun 23;22:498. doi: 10.1186/s12917-026-05672-9 (PMC13520257; doi:10.1186/s12917-026-05672-9)
Supplement: Supplementary file 2 [file 12917_2026_5672_MOESM1_ESM.docx]

**Supplementary Table 1**. Accuracy and Precision Values for LC-MS/MS analysis of amantadine in equine plasma.

| Concentration  (ng/mL) | Intra-day Accuracy (% nominal concentration) | Intra-day Precision  (% relative SD) | Inter-day Accuracy (% nominal concentration) | Inter-day Precision  (% relative SD) |
| --- | --- | --- | --- | --- |
|  |  |  |  |  |
| 0.30 | 96.0 | 10.0 | 102 | 8.0 |
| 80.0 | 98.0 | 8.0 | 109 | 8.0 |
| 500.0 | 99.0 | 7.0 | 100 | 12.0 |
